# CT imaging of and therapy for inflammatory bowel disease *via* low molecular weight dextran coated ceria nanoparticles†

**DOI:** 10.1039/d4nr04994b

**Published:** 2025-03-26

**Authors:** Derick N. Rosario-Berríos, Amanda Y. Pang, Katherine J. Mossburg, Johoon Kim, Víctor R. Vázquez Marrero, Seokyoung Yoon, Mahima Gupta, Olivia C. Lenz, Leening P. Liu, Andrea C. Kian, Kálery La Luz Rivera, Sunny Shin, Peter B. Noël, Elizabeth M. Lennon, David P. Cormode

**Affiliations:** a Department of Biochemistry and Molecular Biophysics, Perelman School of Medicine, University of Pennsylvania Philadelphia PA USA david.cormode@pennmedicine.upenn.edu; b Department of Radiology, University of Pennsylvania, Perelman School of Medicine Philadelphia PA USA; c Department of Bioengineering, University of Pennsylvania Philadelphia PA USA; d Department of Microbiology, University of Pennsylvania, Perelman School of Medicine Philadelphia Pennsylvania USA; e Department of Clinical Sciences and Advanced Medicine, School of Veterinary Medicine, University of Pennsylvania Philadelphia Pennsylvania USA

## Abstract

Inflammatory bowel disease (IBD) affects approximately 3.1 million individuals in the U.S., causing deleterious symptoms such as bloody diarrhea and leading to an increased risk of colorectal cancer. Effective imaging is crucial for diagnosing and managing IBD, as it allows for accurate assessment of disease severity, guides treatment decisions, and monitors therapeutic responses. Computed tomography (CT) with contrast agents is the gold standard for imaging the gastrointestinal tract (GIT). However, current agents are less effective in obese patients and lack specificity for inflamed regions associated with IBD. Moreover, IBD treatments often have limited efficacy and do not address the role of oxidative stress in IBD progression. This study explores dextran-coated cerium oxide nanoparticles (Dex-CeNP) as a CT contrast agent and therapeutic for IBD, leveraging cerium's superior K-edge energy profile, dextran's inflammation-specific targeting, and cerium oxide's antioxidant properties. Herein, we synthesized Dex-CeNP formulations using 5, 10, 25, and 40 kDa dextran to explore the effect of dextran coating molecular weight. *In vitro* assays showed formulation biocompatibility and demonstrated that 5 kDa Dex-CeNP had the highest catalytic activity, which translated into improved suppression of inflammation. As a result, this formulation was selected for *in vivo* use. *In vivo* CT imaging of mice subjected to dextran sodium sulfate (DSS) colitis showed that Dex-CeNP provided better contrast in the GIT of mice with colitis compared to iopamidol (ISO), with pronounced attenuation in the large intestine and disease- specific retention at 24 h. Additionally, Dex-CeNP significantly decreased Disease Activity Index (DAI) scores, and diminished gastrointestinal bleeding when compared with a currently approved drug, indicating that it is an effective treatment for colitis. Studies also revealed that the Dex-CeNPs were safe and well-excreted following administration. In summary, Dex-CeNP has significant promise as a dual-purpose agent for CT imaging and treatment of IBD.

## Abstract 2

This study investigates the synthesis and evaluation of a novel contrast agent, dextran-coated cerium nanoparticles (Dex-CeNP), designed to both image and treat IBD simultaneously.

## Graphical abstract

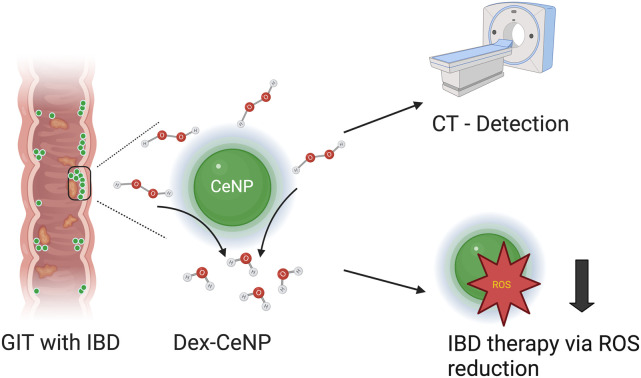

## Introduction

Inflammatory bowel disease (IBD) is characterized by chronic recurrent gastrointestinal tract (GIT) inflammation and affects around 7 million people worldwide with increasing numbers of diagnoses each year.^1,2^ The two main types of IBD are Crohn's disease and ulcerative colitis, both of which cause significant inflammation and damage to the digestive tract.^1,2^ Moreover, IBD reduces life expectancy by five to eight years, depending on demographics.^3^ Existing treatments include anti-inflammatory drugs like aminosalicylates, corticosteroids, immunosuppressants, and biologics that target specific immune pathways. However, these therapies are not curative, often have significant side effects, and become refractory to patients over time, necessitating research into more effective options.^4,5^

Beyond treatment, there is yet to be a single quick, noninvasive tool for diagnosis of IBD. Instead, most patients have to undergo a combination of extensive clinical intake, laboratory workup, endoscopic procedures, and imaging studies.^6–8^ Recently, CT with contrast agents has become the gold standard for diagnosing and tracking IBD over time, rapidly generating multiplanar images for better visualization of the GIT in patients with acute nonspecific abdominal pain.^9,10^ Currently, CT imaging of the GIT is primarily carried out with iodine-based contrast agents.^11^ Although iodinated agents can provide some degree of contrast, the contrast is nonspecific to areas of IBD inflammation.^11,12^ Moreover, since IBD is often marked by gut barrier dysfunction, ulceration, and erosion of the intestinal lining, highlighting these pathological areas on CT could provide significant advantages over existing assessment methods, such as endoscopy, which patients find uncomfortable and invasive.^13,14^ Due to these factors, there is a need for the development of IBD-specific contrast agents.

Nanoparticles have garnered significant attention as CT contrast agents due to the ease of synthesizing them in various sizes and with different coatings, which can target specific disease sites.^15–19^ Cerium oxide nanoparticles (CeNP) present an effective alternative as CT contrast agents, with minimal previous exploration as a contrast agent. Cerium has a superior K-edge at 40.4 keV compared to iodine's 33.2 keV, allowing it to produce stronger CT contrast. Additionally, cerium is less expensive than other elements that have been proposed as CT contrast agents such as gold.^20^ Furthermore, CeNP have been proposed as artificial antioxidants because they neutralize free radicals.^21^ Previous studies have shown that cerium oxide nanoparticles can protect cells from reactive oxygen species (ROS) and improve oxidation-related pathologies.^22,23^ In particular, CeNP can neutralize free radicals through their unique redox cycling ability between the Ce^3+^ and Ce^4+^ oxidation states, allowing them to mimic the activities of superoxide dismutase (SOD) and catalase (CAT). They can also induce strong anti-apoptotic effects and promote wound healing.^23^ This is important since patients with IBD experience increased oxidative stress, with recent studies suggesting that administering antioxidants may benefit IBD treatment due to the tissue damage associated with oxidative stress.^24^

**Fig. 1:**
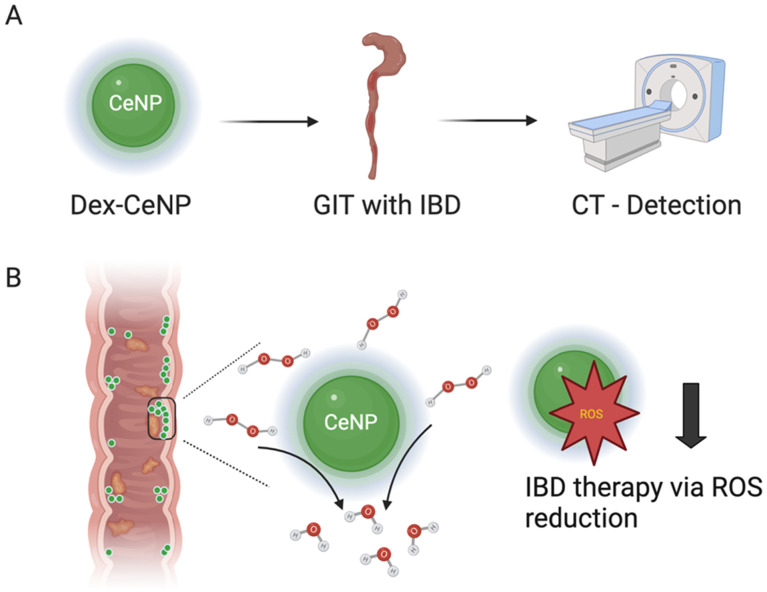
Schematic depictions of Dex-CeNP functions (A) as a contrast agent and (B) as treatment for inflammation in the GIT.

In this study, we develop dextran-coated cerium oxide nanoparticles (Dex-CeNP) for imaging the GIT and as a treatment option for IBD due to their antioxidant properties (Fig. 1). We employ dextran, a polysaccharide composed of glucose molecules that can be obtained at different molecular weights, to provide benefits to cerium oxide nanoparticles^25^ such as stability in aqueous environments, enhanced biocompatibility, and increased specificity for inflammation sites.^26–28^ Dextran can also target macrophages by binding to scavenger receptors and C-type lectins on their surface, facilitating the NP's uptake into these inflammation-associated cells. This targeted approach helps localize the NPs to sites of inflammation, where they can more effectively exert their therapeutic effects.^29,30^

However, currently, the effect of dextran coating molecular weight on cerium oxide catalytic activity is unknown. We herein report the synthesis of Dex-CeNP variants using dextran of different molecular weights (5, 10, 25, and 40 kDa) and compare them *in vitro* to identify the best formulation for contrast generation, safety, and therapeutic effects. Furthermore, we report the effects of administering a selected Dex-CeNP formulation orally in a DSS mouse model of IBD. Orally administered drugs are generally more convenient for patients, reduce the risk of complications associated with intravenous administration, and enhance patient compliance.^31^ Additionally, oral delivery allows for targeted treatment directly at the site of GIT inflammation, potentially improving therapeutic outcomes.^32^ We report the CT imaging characteristics of Dex-CeNP in an IBD model compared with clinically approved iodinated agents. In addition, we report their effects as anti-inflammatory agents compared with a clinically used drug, 5-aminosalicylic acid (ASA), as well as their safety and excretion.

## Methods

### Materials

Cerium(iii) nitrate hexahydrate (99.99%) and ammonium hydroxide (28.0–30.0% NH_3_ basis) were obtained from Sigma-Aldrich (St Louis, MO). Dextran of varying molecular weights (*i.e.* 5, 10, 25 or 40 kDa) was purchased from Pharmacosmos (Morristown, NJ). The SOD colorimetric activity kit, the Amplex red hydrogen peroxide/peroxidase assay kits, and hydrochloric acid solution (36.5 to 38.0% HCl) were purchased from Thermo fisher Scientific (Waltman, MA). Mouse TNF-α ELISA kits were purchased from Biolegend (San Diego, California). RAW 264.7, and C2BBe1 cell lines were purchased from American Type Culture Collection (ATCC) (Manassas, VA).

### Dex-CeNP synthesis

The synthesis of the Dex-CeNP formulations was based on a previously reported ammonium hydroxide precipitation protocol.^20^ 1 mL of 1 M cerium(iii) nitrate hexahydrate was dissolved in 2 mL of 0.05 M dextran solution and agitated through sonication. Subsequently, the cerium nitrate and dextran solution were added dropwise to a magnetically stirring glass vial containing 6 mL of 28–30% ammonium hydroxide (NH_4_OH) and allowed to heat at 90 °C for 1 h. Following the 1 h heating period, the glass vial was covered with aluminum foil containing tiny holes and allowed to stir at room temperature overnight. The Dex-CeNP formulation was first transferred into a Falcon tube and centrifuged for 10 min at 4000 rpm to remove large aggregates. The remaining supernatant was then transferred to ultra-filtration tubes (MWCO 100 kDA) and washed three times with DI water to concentrate the solution. This protocol was used to synthesize all Dex-CeNP variants using dextran of varying molecular weights (*i.e.* 5, 10, 25 or 40 kDa). All concentrated Dex-CeNP formulations were stored at 4 °C prior to further experimentation.

### Characterization

The Dex-CeNP formulations were characterized *via* transmission electron microscopy (TEM, JEOL 1010 electron microscope), dynamic light scattering (DLS, Zetasizer Nano ZS90), ultraviolet-visible spectrometry (UV-Vis, Genesys 150), Fourier-transform infrared spectroscopy (FT-IR, JASCO FT/IR-480 PLUS), and X-ray power diffraction (XRD, Rigaku GiegerFlex D/Max-B). The concentrations of cerium were determined through inductively coupled plasma-optical emission spectrometry (ICP-OES, Spectro Genesis ICP).

### Transmission electron microscope

The size and shape of the Dex-CeNP formulations were confirmed with a JEOL 1010 transmission electron microscope from JEOL USA Inc. (Peabody, MA) set at 80 kV transmission. For each Dex-CeNP formulation, 10 μL of the nanoparticle solution was diluted in 990 μL of water. Then, 10 μL of this diluted nanoparticle solution was placed on carbon-coated copper grids (FCF-200-Cu, Electron Microscopy Services, Hatfield, PA) and allowed to dry prior to imaging. Size was manually measured with imageJ software developed by the National Institutes of Health.

### Hydrodynamic diameter and zeta potential

The hydrodynamic diameters and zeta potential of the Dex-CeNP formulations were determined with a Nano ZA-90 Zetasizer from Malvern Instruments (Worcestershire, UK). Measurements were taken on 1 mL diluted samples (10 μL of Dex-CeNP from stock solution diluted in 990 μL of DI water) and reported as hydrodynamic diameter and the *Z*-average.

### UV-Vis

UV-Vis spectra of the Dex-CeNP formulations were collected using a Genesys 150 UV-visible spectrophotometer from Thermo Scientific (Waltham, MA), with wavelengths ranging from 200 to 800 nm. Absorbance was measured on cuvettes filled with 1 mL diluted samples (10 μL of Dex-CeNP from stock solution diluted in 990 μL of DI water).

### FT-IR

FT-IR spectra of the Dex-CeNP formulations and free dextran were collected using a JASCO FT/IR-480 Plus (Easton, MD) set in transmission mode. Samples were prepared by first grinding each formulation with dry KBr, and subsequently pressing the samples into thin pellets.

### XRD

The XRD pattern of the Dex-CeNP formulations were collected with a Rigaku GiegerFlex D/Max-B X-ray diffractometer set at 45 kV and 30 mA, with radiation wavelength of 1.54. Scans were taken at a rate of 2° per minute, recording the XRD patterns between 20 and 90°.

### ICP-OES

The concentrations of the Dex-CeNP formulations were determined with a Genesis ICP from Spectro Analytical Instruments GmbH (Kleve, Germany). Samples were prepared by dissolving 5 μL of Dex-CeNP stock solutions in 995 μL of aqua regia (1 : 3 ratio of concentrated hydrochloric acid to concentrated nitric acid). After 2 h of complete dissolution of the Dex-CeNP formulations, the final volume was brought up to 6 mL by adding 5 mL of DI water. Cerium concentrations were determined using the average of four measurements per formulation.

### *In vitro* stability

The stability of the Dex-CeNP formulations was tested by incubating each formulation in an acidic environment that simulates gastric conditions. The pH of distilled water was adjusted to 2 using hydrochloric acid and sodium hydroxide. Dex-CeNP were dispersed in that solution at a concentration of 1 mg mL^−1^ and incubated at 37 °C for 2 h. The hydrodynamic diameters of the Dex-CeNP were then determined through DLS and compared to baseline measurements.

### Catalase-mimetic activity assay

The CAT-mimetic activity of Dex-CeNP was assessed using an Amplex Red Hydrogen Peroxide/Peroxidase Assay Kit. Dex-CeNP solutions of various concentrations (0.01, 0.5, and 1.0 mg mL^−1^) were prepared in the reaction buffer. After adding 50 μL of each Dex-CeNP solution to the wells of a 96-well plate, 50 μL of 40 μM hydrogen peroxide (H_2_O_2_) was added. Following a 20 minute incubation, 50 μL of the working solution was added to each well. The mixture was then allowed to react at room temperature for an additional 30 min before measuring the absorbance at 560 nm.

### SOD-mimetic activity assay

The SOD-mimetic activity of Dex-CeNP was assessed using a SOD colorimetric activity kit. Dex-CeNP stock solution was diluted to different concentrations (0.01, 0.05, and 0.1 mg mL^−1^) in PBS. For each concentration, 20 μL was added to the wells of a 96-well plate. Following the kit instructions, the substrate and xanthine oxidase were added to the wells, and the mixture was incubated at room temperature for 20 min. The absorbance was then measured at 450 nm.

### CT phantom imaging

The CT contrast generation of the Dex-CeNP formulations was evaluated with a conventional clinical CT system. Samples of each formulation and ISO controls were prepared at a range of concentrations (0, 0.5, 1, 2, 4, 8, and 12 mg mL^−1^) in triplicate. The samples were subsequently placed into custom made plastic phantoms and submerged in water to realistically represent the human abdomen and beam-hardening effects. To assess the CT contrast generation of each Dex-CeNP against ISO, salt, and water, the samples were scanned using a Spectral CT 7500 scanner (Philips Healthcare, Best, Netherlands) at 80, 100, 120, and 140 kV, a matrix size of 512 × 512, a field view of 37 × 37 cm, and a reconstructed slice thickness of 0.5 mm. The attenuation rates of each formulation were analyzed using OsiriX MD software. *t*-Values were calculated from the attenuation rates and the standard errors of the data compared with the 5% reference *t*-value at six degrees of freedom.

### Cell culture

C2BBe1 (human epithelial colorectal adenocarcinoma) and RAW 264.7 (macrophages) were purchased from ATCC (Manassas, VA). Both cell lines were maintained in a Dulbecco's modified Eagle's medium containing 10% fetal bovine serum (FBS) (Gibco) and 1% penicillin–streptomycin (Thermo Fisher Scientific, Waltham, MA). The cells were incubated at 37 °C in a 5% carbon dioxide (CO_2_) incubator.

### Cell assays (MTS assay, ELISA)

#### *In vitro* cytocompatibility

The *in vitro* cytocompatibility of the Dex-CeNP formulations was assessed in macrophage (RAW 264.7) and enterocyte (C2BBe1) cells due to the likelihood of these cells receiving high exposure to Dex-CeNP upon administration. In brief, 200 μl of media containing 20 000 cells was seeded in each well of the 96-well plate, and the plate was incubated for 24 h at 37 °C in a humidified incubator with 5% CO_2_. Following the 24 h incubation, the cell monolayer was carefully washed with sterile 1× PBS and then treated with Dex-CeNP formulations at concentrations ranging from 0 to 1 mg mL^−1^. After 4 h of exposure, the media was removed and the cell monolayer was washed with sterile PBS.^33^ Cell viability was assessed using the MTS assay (CellTiter 96 cell proliferation assay kit, Promega, Madison, WI) and carried out in 96-well flat bottom microplates (Corning, NY).

#### Antioxidant MTS viability assay

The antioxidant properties of the Dex-CeNP formulations were investigated using RAW 264.7 cells. To evaluate the protective abilities of Dex-CeNP against oxidative damage, 20 000 cells in 200 μL of cell culture media were seeded into each well of a 96-well flat-bottom microplate. After 24 h, the cells were washed with sterile PBS and then incubated with a solution of 0.01% H_2_O_2_ in cell culture media. After 30 min, the cells were washed with sterile PBS before being incubated with different Dex-CeNP formulations for 4 h. Cell viability was determined at the 4 h mark using an MTS assay (CellTiter 96 cell proliferation assay kit from Promega), and the cell viability was normalized to the control.

#### Assessment of TNFα cytokine release *via* ELISA

Levels of the proinflammatory cytokine TNFα realesed by RAW 264.7 macrophages were assessed. The cells were seeded at a density of 1 × 10^6^ cells per well in 96-well plates and cultured overnight. After incubation, the media was removed and replaced with fresh media containing 200 ng mL^−1^ of lipopolysaccharide (LPS) or vehicle. The cells were incubated for 1 h to allow for LPS stimulation. Following this, different concentrations of Dex-CeNP formulation solutions were added to the cell culture media. At the end of the treatment, the media were removed, and TNFα cytokine levels was evaluated using a commercially available ELISA kit (Biolegend, San Diego, California) according to the manufacturer's instructions.

#### Cell uptake study

To investigate the uptake mechanism of Dex-CeNP in RAW 264.7 macrophages, cells were seeded at a density of 1 × 10^6^ cells per well in six-well plates with 2 mL of media per well and cultured overnight. For the inhibition study, phenylarsine oxide (Sigma-Aldrich, St Louis, MO, USA) was added to the cells at a final concentration of 3 μM and preincubated for at least 30 min. After preincubation, Dex-CeNP was added, and the cells were incubated for an additional 4 h.^34^ The treated cells were then washed three times with PBS, harvested using a cell scraper, and centrifuged.^35^ The resulting pellet was dissolved in acid for ICP-OES analysis to quantify Dex-CeNP uptake. This method allowed us to assess the role of clathrin-mediated endocytosis in the internalization of Dex-CeNP by comparing uptake in the presence and absence of phenylarsine oxide.

#### Animal model

The following protocols were approved by the Institutional Animal Care and Use Committee (IACUC) of the University of Pennsylvania under protocol number 806566.

#### DSS-colitis mice model

To induce acute colitis in mice, 7 weeks old male C57BL/6 mice (Jackson Laboratory, Bar Harbor, Maine) were given DSS solution over the course of 8 days using a procedure previously outlined.^36^ On Day 1, mice were weighed and their cage water supply filled with 3% solution of DSS (36 000–50 000 Da, MP Biomedicals, Irvine, California). On both Day 3 and Day 5, the old DSS solution was replaced with a fresh DSS solution. On Day 8, any remaining DSS solution was exchanged for autoclaved water. Throughout this 8 day process, healthy control mice received non-DSS containing water. The weights of the mice was recorded daily to monitor their health and to ensure the proper progression of colitis, as weight loss serves as a key indicator that the assay is working as expected (*n* = 6 per group).

#### *In vivo* imaging

The previously described DSS-colitis and control mice (*n* = 6 per group) underwent *in vivo* imaging studies performed with a MILabs U-CT (MILabs, Utrecht, Netherlands) using a tube voltage of 55 kV and isotropic 100-micron voxels. After baseline CT scans of were taken, 5 kDa Dex-CeNP(250 mg kg^−1^) or ISO (100 mg kg^−1^) were administered *via* gavage. Following the administration of contrast agents, the mice underwent CT scans at 5 min, 30 min, 60 min, 120 min, and 24 h. The images were subsequently analyzed using OsiriX MD software, in which the change in attenuation values between pre- and post-administration time points was measured in key GIT organs like the stomach, small intestine, and large intestine. Additionally, scans were performed on a first-generation dual-source Spectral Photon Counting CT (SPCCT) scanner (NAEOTOM Alpha, Siemens Healthineers, Erlangen, Germany) equipped with two photon-counting detectors. Data acquisition and reconstruction of the phantom were performed utilizing the following parameters: tube voltage 120 kVp, rotation time 0.25 s, collimation 144 × 0.4 mm, slice thickness 0.4 mm, reconstruction filter Qr40, iterative reconstruction QIR 3, matrix size 512 × 512, field of view 205 mm, and pixel spacing (in *x* and *y*) 0.4 mm. In all cases, the obtained images were analyzed using Osirix MD software.

#### Biodistribution

The biodistribution of Dex-CeNP was determined in treated DSS-colitis and healthy mice 24 h after the formulations were administered. In brief, mice were euthanized using anesthesia and cervical dislocation. The heart, lungs, liver, spleen, and kidneys were collected and flushed with PBS. After the mass of each organ was taken, the organs were transferred to glass vials filled with 800 μL of nitric acid and placed into an oven 75 °C overnight to dissolve. Following this initial incubation period, 200 μL of hydrochloric acid was added and allowed to incubate for an additional 3 h. Samples were brought up to 10 mL with DI water and ICP-OES was then run on the samples to quantify the cerium concentration or iodine in each organ.

#### Therapeutics assay

The ability of Dex-CeNP to protect against progression of IBD was assessed in the previously described DSS-colitis mouse model. The following groups were used (*n* = 6 per group): (1) vehicle control with DSS-colitis (2) 5 kDa Dex-CeNP with DSS-colitis (3) 5-ASA with DSS-colitis (treatment control). In all groups, the treatments were administered *via* oral gavage with a concentration of 250 mg kg^−1^ for 5 kDa Dex-CeNP and 100 mg kg^−1^ for 5-ASA. Throughout treatment, mice were weighed every day and fecal occult blood testing was conducted. After five days of treatment, the mice were sacrificed and dissected. The colon length and weight were recorded. To determine the severity of the IBD, the weight and length of each colon was measured using an established protocol.^37^ In addition, the DAI was calculated as described by Iwao *et al.*, *i.e.*, the sum of the weight loss, diarrhea, and bleeding, resulting in a score ranging from 0 (unaffected) to 12 (severe IBD).^38^

#### Histology

Histological studies were carried out on stomach, small intestine, and large intestine tissue samples. After the organs were harvested, the stomach and small intestine were washed with PBS, cut into small pieces, fixed in 10% neutral buffered formalin, dehydrated with ethanol, embedded in paraffin, and sectioned. The samples were stained with hematoxylin and eosin (H&E) before being imaged by Leica DM4000B upright microscope equipped with a Spot RT/SE slider camera. The entire length of the large intestine was also assessed *via* histology, by use of the Swiss roll technique, in which the large intestine is cut along its length, rolled up, fixed in the same manner as the small intestine, embedded, and sectioned. The colonic disease severity was scored by a blinded veterinary pathologist using a standardized scoring system.^39,40^ This scoring evaluates various aspects of tissue damage and inflammation, including DSS/mucosal/crypt loss, crypt inflammation, lamina propria mononuclear cells, neutrophils, epithelial hyperplasia, and edema/fibrosis. Each category is scored on a scale from 0 to 3 or 4, with higher scores indicating more severe pathology. The maximum possible total score is 19, reflecting the cumulative severity of tissue changes (ESI Table S8†).^39,40^ Furthermore, the kidneys and liver were harvested, rinsed with cold PBS, and sliced into 5 mm thick sections. The organ samples were fixed in 10% buffered formalin overnight at 4 °C. Following fixation, the samples were processed and stained with hematoxylin and eosin (H&E) by the Pathology Core at the Children's Hospital of Philadelphia.

#### Clinical blood chemistry

Whole blood samples (150 μL) were obtained from the mice *via* cardiac puncture. The blood was left at room temperature for 30 min to clot. The clots were removed by centrifugation at 1000*g* for 10 minutes, and the resulting serum was collected and stored at −20 °C until analysis. Serum biomarkers were evaluated by IDEXX BioAnalytics (North Grafton, MA). Data were expressed as mean ± SEM.

#### Statistical methods

All statistical analyses were performed using GraphPad Prism 7 software (GraphPad Prism Software Inc., San Diego, California). In this study, error bars in the figures represent the standard error of the mean. Table 1 presents the mean and standard deviation for the core diameter, hydrodynamic diameter, and zeta potential of various Dex-CeNP formulations. Statistical analysis for histology and clinical blood chemistry was performed using Tukey's multiple comparisons test with GraphPad Prism. To analyze the interactions between Dex-CeNP in terms of cerium content distribution in organs, a Tukey's multiple comparisons test was conducted.

**Table 1:** Physicochemical properties of Dex-CeNP synthesized with varying dextran molecular weights

Dex-CeNP coating MW	Core diameter (nm)	Hydrodynamic diameter (nm)	Zeta-potential (mV)	Absorbance peak (nm)
5 kDa	4.2 ± 0.9	12.5 ± 0.5	−0.5 ± 1.3	290
10 kDa	3.5 ± 0.8	14.1 ± 2.3	2.8 ± 0.7	290
25 kDa	3.5 ± 0.6	12.6 ± 2.5	−0.2 ± 0.6	290
40 kDa	3.6 ± 0.7	9.6 ± 0.8	0.6 ± 0.5	290

## Results

### Synthesis and characterization

Dex-CeNP were synthesized *via* precipitation with ammonium hydroxide.^20^ Various Dex-CeNP formulations were created using dextran of different molecular weights (5, 10, 25, and 40 kDa) and were characterized using TEM, XRD, zeta potential, DLS, ICP-OES and FT-IR. Dextran was chosen as the NP coating for its stability in biological settings and its ability to allow access of ROS to the cerium oxide surface for neutralization.^20^ Importantly, dextran is an FDA-approved polymer, enhancing its clinical translation potential.^41^ Moreover, activated macrophages in inflammatory sites actively uptake dextran NPs *via* binding to macrophage scavenger receptors, potentially improving specificity to sites of inflammation.^30^ The variations in dextran molecular weight were expected to influence hydrodynamic size and catalytic activities of the NPs, thereby affecting biological efficacy. We performed this synthesis in the absence of dextran and found the resulting nanoparticles to be highly unstable towards aggregation in storage conditions, highlighting the value of the dextran coating.

**Fig. 2:**
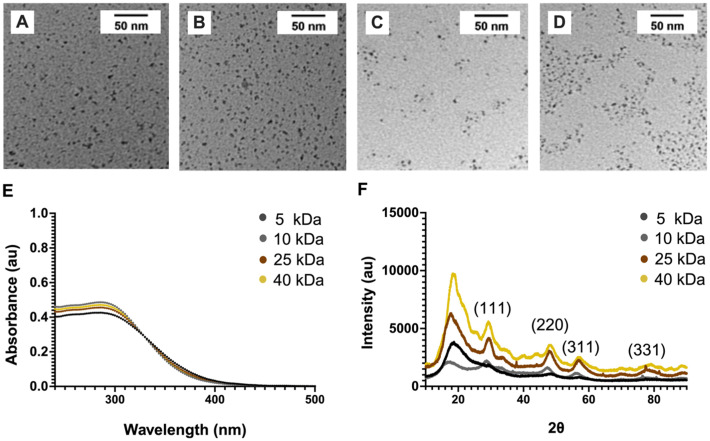
Characterization data of Dex-CeNPs. TEM of Dex-CeNP whose dextran was (A) 5 kDa, (B) 10 kDa, (C) 25 kDa, or (D) 40 kDa in molecular weight. The scale bars represent 50 nm in all panels. (E) UV-Vis spectra of Dex-CeNP dispersed in water. (F) XRD data from the Dex-CeNP.

TEM micrographs revealed well-dispersed, roughly spherical shapes with low size variation (Fig. 2A–D). As summarized in Table 1, we found the core sizes of these NPs to be around 4 nm, as derived from the TEM micrographs. Table 1 also lists the hydrodynamic diameters, which were slightly larger than the core diameters, a common observation for these types of NPs.^23^ There was no clear trend in the hydrodynamic diameters found. Zeta potential measurements showed a close to neutral charge. Characteristic absorption peaks at 290 nm were found in UV-Vis spectra for all formulations (Fig. 2E).

FT-IR analysis confirmed the presence of the dextran coating in all NP formulations, with characteristic peaks of dextran in the Dex-CeNP spectrum closely matching those of free dextran (Fig. S1†). Notably, the FT-IR spectra displayed intense peaks at 3392 cm^−1^ for O–H stretching of hydroxyl groups, 2924 cm^−1^ for C–H bond stretching, and 1153 cm^−1^ for C–O–C vibration of the glycosidic bridge between saccharide units.^20^ Additionally, all Dex-CeNP formulations were evaluated using powder XRD. The XRD patterns showed characteristic diffraction peaks of cerium oxide nanocrystals (Fig. 2F). The cerium oxide cores in Dex-CeNP were identified as face-centered cubic (FCC) crystals, indicated by the presence of (111), (220), (311), and (331) planes (JCPDS # 34-0394).^42,43^ Note that the peaks observed are quite broad due to the small size of the Dex-CeNP cores.^44^ These results are consistent with those previously obtained for the 10 kDa Dex-CeNP formulation.^20^ Moreover, every batch of NPs synthesized underwent size characterization with TEM and UV-Vis as a quality assurance measure.

### Stability and catalytic activities of Dex-CeNP

**Fig. 3:**
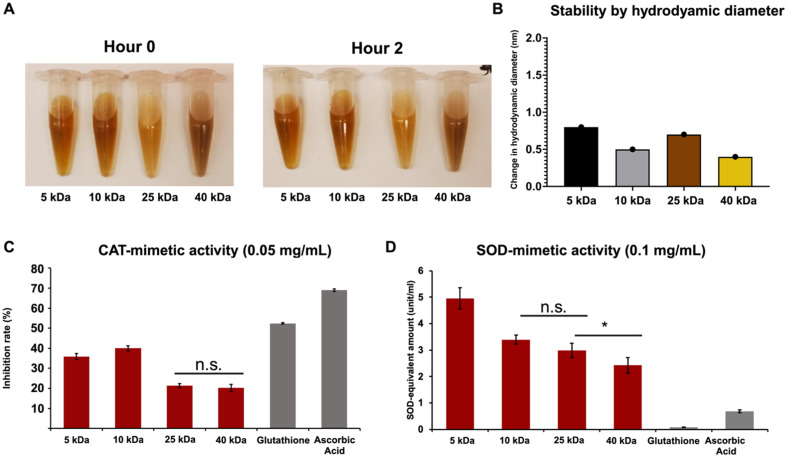
*In vitro* analysis of the Dex-CeNP formulations. (A) Representative images of the formulations before and after 2 h. (B) Formulation stability assessment *via* hydrodynamic diameter measurement using DLS. (C) CAT and (D) SOD-mimetic like activity of the different Dex-CeNP formulations. Error bars represent standard error of the mean. *p* < 0.005 unless indicated otherwise. *p < 0.05. All P values, including significant ones, can be found in the ESI Tables S1 and S2.†

The stability of the NPs in a biologically acidic environment was tested by incubating them for 2 h in a pH 2 solution, similar to that found in the human stomach. The hydrodynamic diameter of the NPs was measured using DLS before and after incubation and no change in size or appearance was observed (Fig. 3A and B). The effects of different dextran molecular weights on catalytic properties, *i.e.* CAT and SOD-mimetic activities, were also studied. These experiments were designed to assess whether altering dextran size could optimize the NPs’ antioxidant potential, which is crucial for therapeutic applications targeting ROS-related pathologies. In general, there was a trend to higher catalytic activity with lower molecular weight dextran, although this was more pronounced for SOD-like activity than CAT-mimetic activity (Fig. 3C, D and S2†). For Fig. 3C and D, the selected concentrations (0.05 mg mL^−1^ for CAT and 0.1 mg mL^−1^ for SOD-mimetic activity) provided the best dynamic range. This observed trend is intuitive, since it might be expected that a shorter polymer would render the inorganic surface more accessible to reagents, thereby elevating catalytic activity.^45^ Importantly, this outcome also confirms that these NPs possess significant antioxidant capabilities through both CAT and SOD activities, underscoring their potential effectiveness in neutralizing ROS and offering therapeutic benefits in oxidative stress-related conditions.^46,47^

### Dex-CeNP provides superior CT contrast

**Fig. 4:**
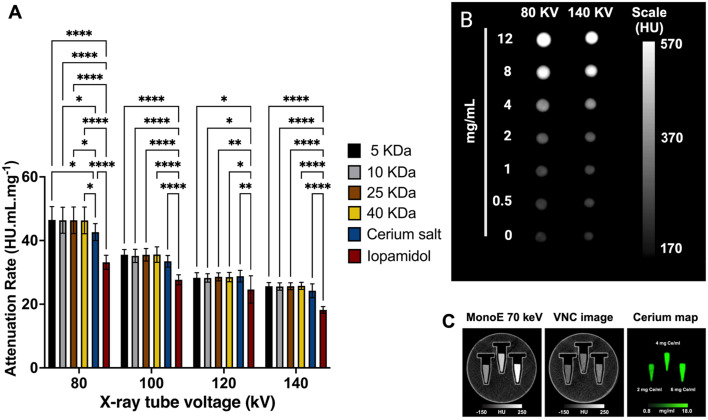
Contrast generation scanned with a clinical CT system of a phantom composed of varying concentrations of Dex-CeNP. (A) Attenuation Rates for Dex-CeNP formulations obtained from CT scans at different X-ray tube voltages: 80, 100, 120, and 140 kV. Error bars represent the standard error of the mean, with asterisks indicating statistical significance levels: *P < 0.05, **P < 0.01, and ****P < 0.0001. (B) Representative CT images of a phantom containing 5 kDa Dex-CeNP (80 kV vs 140 kV). (C) SPCCT images of Dex-CeNP samples. Left to right: MonoE 70 keV image, virtual non-contrast (VNC) image and a cerium material map.

The contrast agent performance of Dex-CeNP was determined by measuring their contrast generation in a clinical CT system using a custom-built phantom comprised of materials with similar properties to GIT tissue.^48^ Samples were prepared in triplicate with a range of concentrations. The contrast generated by these formulations was compared to that of ISO, which is commonly used as contrast in current clinical settings, and cerium nitrate salts. To assess the conventional CT contrast properties of Dex-CeNP, samples were scanned using a Spectral CT 7500 scanner at 80, 100, 120, and 140 kV. All Dex-CeNP formulations demonstrated strong CT contrast generation (Fig. 4A and B) with no differences observed between the different coating molecular weights at the X-ray tube voltages used. We anticipated this, because the atomic weights of the elements that compose dextran are similar to that of water and therefore should not generate contrast in CT. In addition to this, since all the formulations share the same core element (cerium), we would expect their *in vitro* contrast performance to remain consistent regardless of the coating molecular weight. Thus, the dextran coating plays no role in altering CT contrast. Additionally, it was also found that all Dex-CeNP formulations produced greater attenuation than ISO at all X-ray tube voltages as reported in our previous studies.^20,49^ This is due to iodine having a lower k-edge value (33.2 keV) compared to that of cerium (40.4 keV), so iodine's X-ray attenuation peak is consistently further from the average beam energy. Futhermore, a phantom scan of Dex-CeNP using a clinical SPCCT (Fig. 4C) demonstrated that cerium can be specifically imaged and virtual non-contrast maps can be generated, which are comparable to traditional non-contrast scans.^50^

### Dex-CeNP are cytocompatible and protect against ROS *in vitro*

**Fig. 5:**
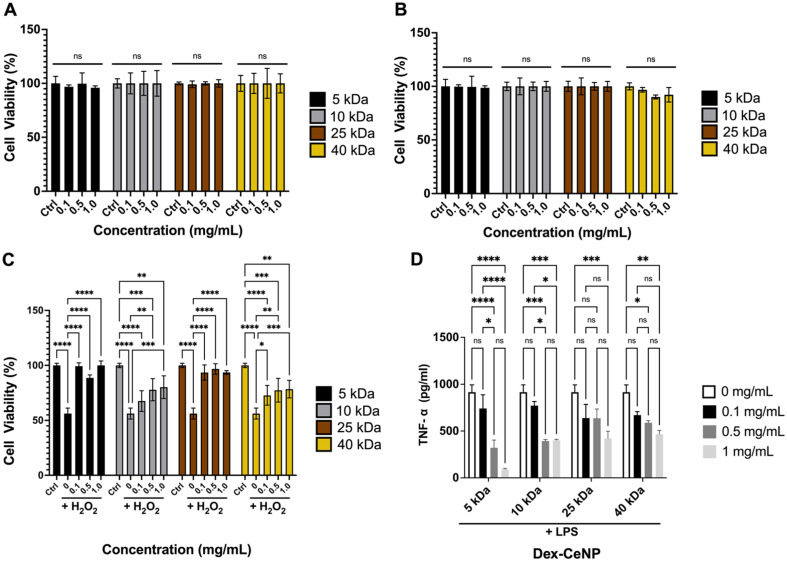
*In vitro* assays. (A) Effect of Dex-CeNP formulations on the viability of RAW 264.7 and (B) C2BBe1 as determined by the MTS assay. *In vitro* assessment of anti-inflammatory activity by (C) MTS assay and (D) TNF-α ELISA using RAW 264.7 cells. Error bars represent the standard error of the mean, with asterisks indicating statistical significance levels: *P < 0.05, **P < 0.01, and ****P < 0.0001. All P values, including significant ones, can be found in the ESI Table S3–S6.†*n* = 6 per group.

The cytocompatibility of Dex-CeNP in colon epithelial (C2BBe1) and macrophage (RAW 264.7) cell lines was evaluated to understand their potential response to Dex-CeNP upon administration. Cytocompatibility was assessed by incubating NPs for 4 h at various Dex-CeNP concentrations (0 to 1 mg Ce per mL) and conducting a cell viability assay. These concentrations were chosen to cover the expected *in vivo* exposure range. Notably, no significant changes in cell viability were observed for any of the formulations in either cell line. Our data therefore strongly supports the cytocompatibility of Dex-CeNP across all formulations (Fig. 5A and B).

We investigated the anti-inflammatory properties of Dex-CeNP in RAW 264.7 due to their crucial role in inflammatory conditions such as IBD.^51^ Cells were incubated with vehicle, Dex-CeNP alone, H_2_O_2_, or Dex-CeNP/H_2_O_2_. Encouragingly, all Dex-CeNP formulations improved cell viability after peroxide treatment (Fig. 5C). In addition, we found that the 5 kDa Dex-CeNP formulation best recovered cell viability and at the lowest concentration. This finding is in agreement with the higher catalytic activity of this formulation (Fig. 3). Furthermore, we examined cytokine release (TNF-α) as a marker of inflammatory response *via* an ELISA. These experiments were conducted with an LPS challenge for 1 h prior to Dex-CeNP formulation addition. We found that all formulations reduce TNF-α levels after LPS treatment (Fig. 5D), and that the 5 kDa dextran formulation reduced TNF-α levels more than any other formulation. Given the heightened catalytic and anti-inflammatory effects of the 5 kDa Dex-CeNP formulation, we prioritized it for *in vivo* testing to optimize resource use and minimize animal involvement while focusing on the most promising candidate for therapeutic and imaging applications. Additionally, to study the mechanism of Dex-CeNP uptake in macrophages, incubations were performed using phenylarsine oxide, a clathrin-mediated endocytosis inhibitor. The results, shown in Fig. S3,† demonstrate that phenylarsine oxide suppresses Dex-CeNP uptake, supporting the concept that Dex-CeNP are internalized *via* a receptor-mediated process.

### GIT imaging with Dex-CeNP

We evaluated the *in vivo* CT contrast generation of 5 kDa Dex-CeNP in a DSS colitis mouse model and in control mice. The mice were randomly assigned to receive doses of either water (vehicle control), 5 kDa Dex-CeNP, or ISO *via* gavage and scanned with micro-CT. Scans were done at pre-administration, 5 min, 30 min, 1 h, 2 h, and 24 h post-administration. We selected these time points as they are clinically relevant, and our preliminary studies suggested that most NPs would be excreted within 24 h. Dex-CeNP or ISO were given at a dose of 250 mg Ce or I per kg body weight, a common dosage for X-ray contrast agents,^52,53^ with all treatments being volume-matched (Fig. S4A and S4B†).

**Fig. 6:**
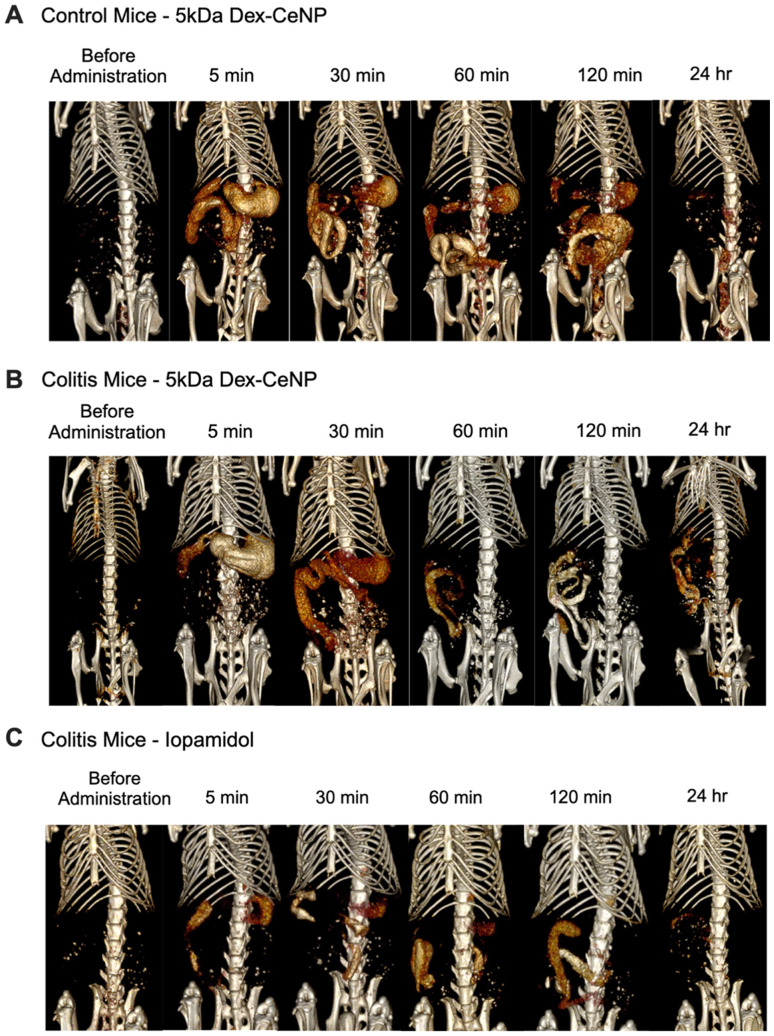
Representative CT images of healthy and DSS colitis mice, before oral administration of CT contrast agents and for 24 h after. (A) Healthy control mice with 5 kDa Dex-CeNP, (B) DSS colitis mice with 5 kDa Dex-CeNP, and (C) DSS colitis mice with ISO.

We used the following groups: colitis mice treated with 5 kDa Dex-CeNP, healthy control mice treated with 5 kDa Dex-CeNP, and colitis mice treated with ISO. *In vivo* CT imaging revealed that Dex-CeNP generated higher contrast in the GIT of mice with induced colitis than in any other group. Notably, there was pronounced attenuation in the large intestine over time and specific retention at 24 h, confirming the efficacy of Dex-CeNP as contrast agents for IBD (Fig. 6).

**Fig. 7:**
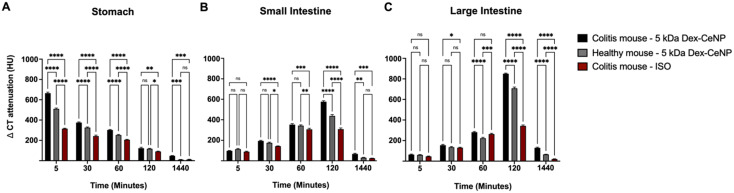
Evaluation of Dex-CeNP as a contrast agent. Time-dependent attenuation variations in various organs including (A) stomach, (B) small intestine, and (C) large intestine. Error bars represent the standard error of the mean, with asterisks indicating statistical significance levels: *P < 0.05, **P < 0.01, and ****P < 0.0001.

Analysis of the micro-CT images showed the change in CT attenuation (HU) over time in the stomach, small intestine, and large intestine across the three experimental groups. Significant differences were observed in the stomach at early time points (5, 30, 60 min), particularly between the colitis mice treated with 5 kDa Dex-CeNP and the other groups, with differences diminishing by 24 h (Fig. 7A). In the small intestine, no significant differences were noted at 5 min, but significant differences emerged at 30 min and persisted through 120 min, with ongoing significance at 24 h (Fig. 7B). In the large intestine, significant differences were initially absent, but emerged at 60 min and became significant at 120 and 24 h (Fig. 7C). Furthermore, the biodistribution data from ICP did not show significant levels of Dex-CeNP in other organs, supporting the notion that oral administration bypasses these organs (Fig. S5†).

### Dex-CeNP are protective in a model of IBD

We tested the ability of 5 kDa Dex-CeNP to protect against the progression of IBD in a DSS mouse model by assessing DAI, biomarkers, and histology. For these experiments, we used the same DSS model described previously. The following groups were used (each with colitis): (1) water control, (2) 250 mg kg^−1^ 5 kDa Dex-CeNP, and (3) 100 mg kg^−1^ 5-ASA (a drug approved for clinical treatment of IBD as a positive treatment control).^54^ The treatments were administered to the animals *via* gavage for five consecutive days to examine the effects of Dex-CeNP during an acute episode (Fig. S6†).

**Fig. 8:**
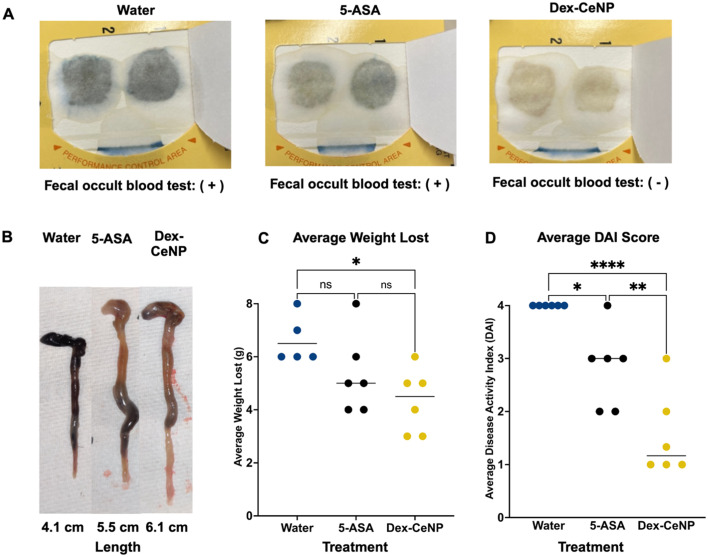
Efficacy of Dex-CeNP as a treatment for IBD. (A) Fecal occult blood test results for mice treated with 5 kDa Dex-CeNP, 5-ASA, and water. (B) Comparison of colon length for mice treated with 5 kDa Dex-CeNP, 5-ASA, and water (*n* = 6). (C) Quantitative results for average weight lost and (D) DAI across different treatment groups, with statistical significance markers. Error bars represent the standard error of mean, with asterisks indicating statistical significance levels: *P < 0.05, **P < 0.01, and ****P < 0.0001.

**Fig. 9:**
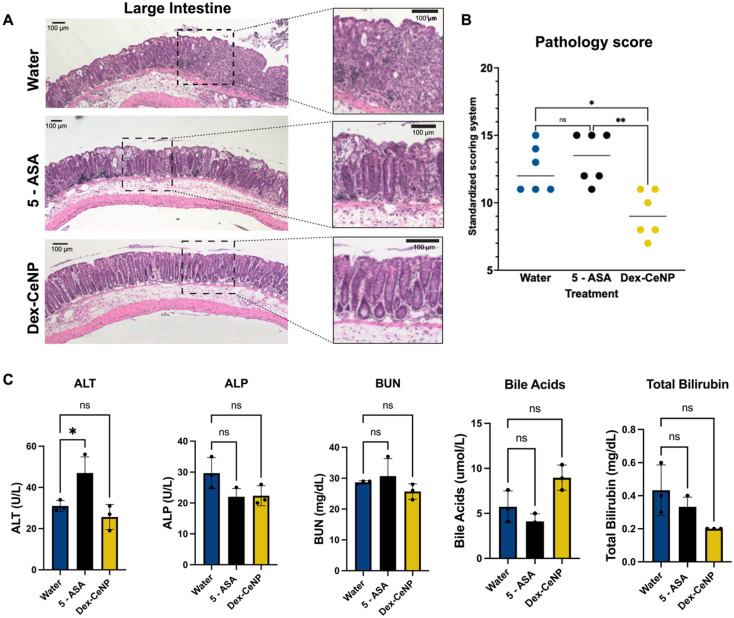
Dex-CeNP is an effective treatment for mice with acute DSS colitis. (A) Histological analysis of large intestines in colitis mice 24 h after Dex-CeNP, 5-ASA or water administration (the left micrographs were taken at 5x magnification, and the insets are magnifications of the boxed areas). (B) Histological scoring of disease severity by a blinded veterinary pathologist using a standardized system. (C) Serum biomarkers for liver and kidney function were assessed in mice 24 h after injection with either the water control, 5-ASA, or Dex-CeNP (*n* = 3 per group).

Fecal occult blood testing was performed on stool samples prior to euthanizing the mice, and their weights were recorded daily. The fecal occult blood testing showed blood in the stool for the water control group and the 5-ASA group, but not for the 5 kDa Dex-CeNP group (Fig. 8A). After five days of treatment, the mice were sacrificed and dissected. In patients with ulcerative colitis, scarring in the colon can lead to a narrowing of the passage, known as a colonic stricture. The length of the colon was slightly shorter in the water control and 5-ASA groups compared to the 5 kDa Dex-CeNP group, although this difference was not statistically significant (*p* = 0.115) (Fig. 8B and ESI Table S7†). Furthermore, mice in the 5 kDa Dex-CeNP group experienced significantly less weight loss compared to water control mice (Fig. 8C). The therapeutic efficacy of Dex-CeNP was highlighted by a marked decrease in DAI scores among Dex-CeNP-treated mice compared to the control group and the conventional treatment (5-ASA) (Fig. 8D). Histological analysis of the large intestine showed a marked improvement in tissue architecture in the Dex-CeNP-treated group compared to the water and 5-ASA groups (Fig. 9A). The Dex-CeNP group exhibited a more intact epithelial layer and reduced cellular infiltration, suggesting effective mitigation of inflammation and preservation of colon structure. The disease severity was scored using a standardized scoring system.^39,40^ The disease score showed similar results to the DAI, where Dex-CeNP showed therapeutic efficacy and treated mice exhibited reduced inflammation in the colon (Fig. 9B). Moreover, we also examined kidney and liver histology, in which there was no apparent changes in any of the groups (Fig. S7 and S8†). Additionally, biochemical markers, including ALT, ALP, BUN, bile acids, and total bilirubin, were assessed, with no significant differences observed between the Dex-CeNP group and the water control treatment group. This finding suggests that Dex-CeNP does not adversely impact liver function. The consistent levels across these markers further indicate that Dex-CeNP treatment does not introduce additional systemic stress (Fig. 9C).

## Discussion

We successfully synthesized and characterized Dex-CeNP with dextran of varying molecular weights (5, 10, 25, and 40 kDa). *In vitro* tests using a clinical CT system indicated that Dex-CeNP consistently delivered strong contrast across various dextran molecular weights, with higher attenuation rates particularly noticeable at lower tube voltages (80 kV). Dex-CeNP showed greater X-ray attenuation than ISO across all tested tube voltages (80 to 140 kV). Cytocompatibility tests with C2BBe1 colon epithelial cells and RAW 264.7 macrophages showed no significant reduction in cell viability across a range of Dex-CeNP concentrations (0 to 1 mg Ce per mL), supporting their potential safety for clinical applications. Additionally, Dex-CeNP demonstrated protective effects against H_2_O_2_-induced oxidative stress and exhibited anti-inflammatory properties by reducing TNF-α production in macrophages, with the 5 kDa Dex-CeNP formulation performing best. Thus, this formulation was selected for *in vivo* studies. Using a DSS-induced colitis mouse model, we found that 5 kDa Dex-CeNP could be effectively used as CT contrast agents *via* significant enhancement in gastrointestinal contrast of colitis mice compared to healthy mice and colitis mice that were administered ISO. We believe this enhanced contrast in diseased gastrointestinal tissue is due to the inflammation in the DSS colitis mice, which causes epithelial damage and increases macrophage levels. The NPs, with their dextran coating, have an affinity towards sites of inflammation. Therefore, we suggest that the Dex-CeNPs preferentially accumulate at these inflamed areas. Additionally, the epithelial damage caused by inflammation may promote transient movement of the NPs across the damaged tissue, further enhancing their localization at the site of inflammation. Importantly, CT images revealed sustained attenuation in the large intestine over a 24 h period, indicative of prolonged retention at the inflammation site. This can result in the accurate identification of inflammation sites, making the detection of IBD easier and more efficient. The robust anti-inflammatory and antioxidant properties of Dex-CeNP, as shown in our *in vivo* studies, suggest that these NPs could be a promising treatment for IBD. These results collectively suggest that 5 kDa Dex-CeNP have significant potential as a dual-function agent for both imaging and therapy, offering a substantial improvement over current diagnostic and therapeutic methods for IBD.

Our research focused on different dextran coatings reveals significant variations in catalytic performance and biological effects. Notably, the 5 kDa Dex-CeNP outperformed other formulations in several key areas. This formulation exhibited higher catalytic activity, particularly in mimicking SOD and CAT activities, which are crucial for neutralizing ROS. The 5 kDa Dex-CeNP also provided better protection against oxidative damage, as demonstrated by their superior ability to mitigate H_2_O_2_-induced oxidative stress compared to higher molecular weight coatings. The 5 kDa formulation was particularly effective in regulating TNF-α levels after LPS stimulation, an important factor since TNF-α is a key inflammatory cytokine involved in the pathogenesis of diseases like IBD.^55^ The higher catalytic activity of Dex-CeNP coated with lower molecular weight dextran is intuitive, since one might expect that a lower molecular weight polymer would afford easier access to the cerium oxide surface.

Our work and that of others on cerium NPs indicate that they may be viable as antioxidant and anti-inflammatory agents. For example, citrate-coated cerium NPs effectively reduced edema and suppressed macrophage recruitment in a mouse model of acute hind paw inflammation.^23^ Furthermore, interests in CeNP for neurodegenerative diseases have increased due to their ability to alter signaling pathways and scavenge ROS.^56,57^ Additionally, nanoceria predominantly accumulated in tumors and significantly decreased tumor growth and volume in tumor-bearing mice over four weeks, demonstrating potential anti-cancer properties against fibrosarcoma.^58,59^

In the past, we and others have studied Dex-CeNP coated with 10 kDa dextran as a CT contrast agent and observed that cerium NPs exhibit anti-inflammatory effects.^20,60–62^ Building on this, we investigated the impact of dextran molecular weight on cerium NPs in this study. Changing the molecular weight of dextran resulted in NPs with remarkably consistent physical characteristics and CT contrast generation. We found that lower molecular weight coatings better protected against ROS, mitigated the inflammatory response, and exhibited higher catalytic activities. Moreover, the 5 kDa Dex-CeNP outperformed ISO, a commonly used contrast agent in clinical settings, and 5-ASA, a conventional treatment for IBD. Previously, Cao *et al.* demonstrated that CeNP were effective in treating animal models of IBD by downregulating pro-inflammatory and fibrosis-related cytokines.^63^ While their research focused on the impact of CeNP on cytokine levels, we found effects on clinically relevant metrics such as histology and DAI scores.

Moving forward, we will explore different administration routes and determine the ideal dosage. We hypothesize that for therapeutic purposes, a lower dose administered throughout the day will be more effective.^64^ Therefore, we aim to assess the effects of Dex-CeNP on colitis by administering this formulation in drinking water. Future work should focus on long-term *in vivo* studies to fully understand the biodistribution, retention, and potential side effects of these NPs. Additionally, we acknowledge that a more comprehensive evaluation, including histopathological analysis, could provide deeper insights into the long-term effects of Dex-CeNP on liver tissue and other relevant organs.^65^ Investigating the efficacy of Dex-CeNP in different models of IBD and other inflammatory conditions could broaden their therapeutic applicability. Given the shared inflammatory microenvironment between IBD and certain cancers, Dex-CeNP could also be adapted for cancer imaging and therapy, extending their utility beyond IBD treatment. In particular, inflammation is a common feature in both IBD and cancer, particularly in tumors such as colon and cervical cancer, where oxidative stress and immune cell infiltration play key roles.^66^

One limitation of this study is the reliance on a single colitis model, which does not fully capture the complexity of human IBD. Moreover, while *in vitro* and short-term *in vivo* results are promising, further investigation of long-term outcomes and safety in humans is needed. Future research should also explore the mechanisms underlying the anti-inflammatory and antioxidant properties of Dex-CeNP to better tailor their use in clinical settings. Lastly, there remains a critical challenge in scaling up the production of Dex-CeNP and ensuring their reproducibility and quality control for potential clinical use that needs to be addressed.

## Conclusion

Dex-CeNP can be synthesized with varying dextran molecular weights (5, 10, 25, and 40 kDa), resulting in consistent particle size and stability in simulated gastric conditions. Dex-CeNP provided superior CT contrast compared to ISO, especially at lower tube voltages, making them effective for enhanced imaging. *In vitro* tests confirmed that Dex-CeNP are highly biocompatible with macrophages and colon epithelial cells, exhibiting strong antioxidant properties and significant anti-inflammatory activity, particularly in reducing TNF-α production. The 5 kDa formulation showed superior performance in these measures and was therefore selected for *in vivo* testing. In a DSS-induced colitis mouse model, 5 kDa Dex-CeNP significantly enhanced gastrointestinal contrast, showed prolonged retention at inflammation sites, and reduced gastrointestinal bleeding, weight loss, and DAI scores. These findings indicate that Dex-CeNP, particularly the 5 kDa formulation, offer promising dual-function capabilities for imaging and treating IBD, providing both diagnostic and therapeutic benefits.

## Author contributions

The project was designed by D.N.R-B., E.M.L., and D.P.C. D.N.R-B., A.P., K.J.M., J.K., V.R.V.M., S.Y., M.G., O.C.L., L.P.L., A.C.K., K.L.L.R., E.M.L., and D.P.C. performed the experiments. D.N.R-B., A.P., S.S., P.B.N., E.M.L., and D.P.C. contributed to data analysis. D.N.R-B and D.P.C. wrote the manuscript. All authors contributed to and have approved the final version of the manuscript.

## Data availability

The authors confirm that the data supporting the findings of this study are available within the article and its ESI.†

## Conflicts of interest

There are no conflicts to declare.

## Supplementary Material

NR-017-D4NR04994B-s001
