# Supplementary material for: CT imaging of and therapy for inflammatory bowel disease via low molecular weight dextran coated ceria nanoparticles
Source: Nanoscale. 2025 Mar 26;17(16):10356–70. doi: 10.1039/d4nr04994b (PMC11967712; doi:10.1039/d4nr04994b)
Supplement: NR-017-D4NR04994B-s001 [file NR-017-D4NR04994B-s001.pdf]

Supplementary Information (SI) for Nanoscale.
This journal is © The Royal Society of Chemistry 2025

CT imaging of and therapy for inflammatory bowel disease via low molecular weight dextran coated ceria nanoparticles

Derick N. Rosario-Berríos,[1] Amanda Y. Pang,[2] Katherine J. Mossburg,[2,3] Johoon Kim,[2,3] Víctor R. Vázquez Marrero,[4] Seokyoung Yoon,[2] Mahima Gupta,[2] Olivia C. Lenz,[5] Leening P. Liu,[2,3] Andrea C. Kian,[2,3] Kálery La Luz Rivera,[1] Sunny Shin,[4] Peter B. Noël,[2,3] Elizabeth M. Lennon,[5] and David P. Cormode[1,2,3*]

[1]Department of Biochemistry and Molecular Biophysics, Perelman School of Medicine, University of Pennsylvania, Philadelphia, PA, USA

[2]Department of Radiology, University of Pennsylvania, Perelman School of Medicine, Philadelphia, PA, USA

[3]Department of Bioengineering, University of Pennsylvania, Philadelphia, PA, USA

[4]Department of Microbiology, University of Pennsylvania, Perelman School of Medicine, Philadelphia, Pennsylvania, United States of America

[5]Department of Clinical Sciences and Advanced Medicine, School of Veterinary Medicine, University of Pennsylvania, Philadelphia, Pennsylvania, USA

*Corresponding author

E-mail: david.cormode@pennmedicine.upenn.edu

[1]Department of Biochemistry and Molecular Biophysics, Perelman School of Medicine, University of Pennsylvania, Philadelphia, PA, USA

[2]Department of Radiology, University of Pennsylvania, Perelman School of Medicine, Philadelphia, PA, USA

[3]Department of Bioengineering, University of Pennsylvania, Philadelphia, PA, USA

[4]Department of Microbiology, University of Pennsylvania, Perelman School of Medicine, Philadelphia, Pennsylvania, United States of America

[5]Department of Clinical Sciences and Advanced Medicine, School of Veterinary Medicine, University of Pennsylvania, Philadelphia, Pennsylvania, USA

*Corresponding author

E-mail: david.cormode@pennmedicine.upenn.edu

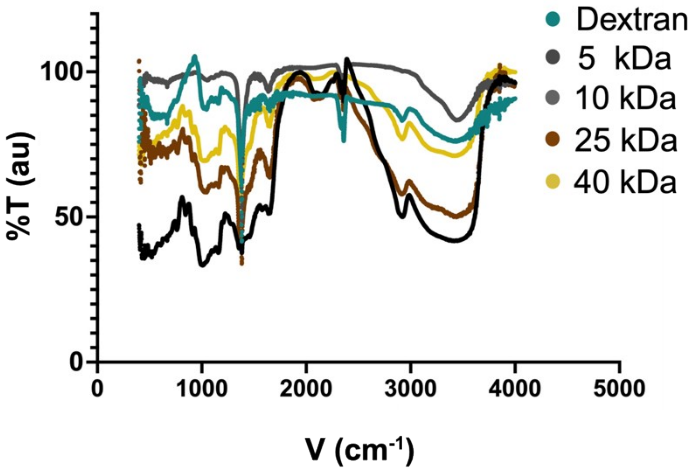

Figure S1: FTIR spectra of the Dex-CeNP formulations and free dextran.

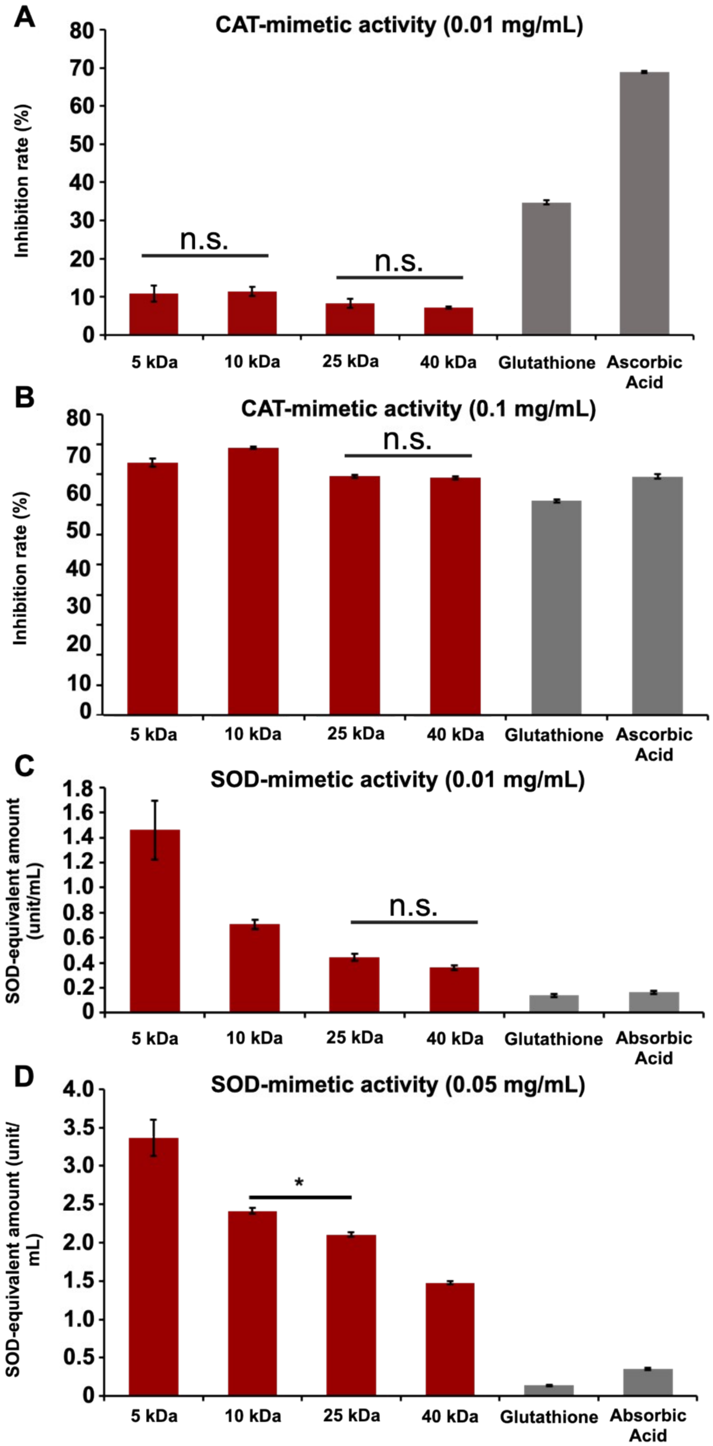

Figure S2: Comparative analysis of CAT-mimetic and SOD-mimetic activities in different Dex-CeNP formulations and antioxidants. CAT-mimetic activity at (**A**) 0.01 mg/mL and (**B**) 0.1 mg, respectively, showing the inhibition rate (%) for various Dex-CeNP formulations compared to

glutathione and ascorbic acid. SOD-mimetic activity at (**C)** 0.01 mg and (**D**) 0.05 mg, respectively, showing the SOD-equivalent amount (units/mL) for the same formulations compared to glutathione and ascorbic acid. $p < 0.005$ unless indicated otherwise. * = $p < 0.05$. (mean ± SEM). All P values, including significant ones, can be found in the **Supplemental Table S1 and S2**.

**Tukey's multiple comparisons test**		
**0.01**	**P Value**	**Summary**
5 kDa vs 10 kDa	0.90906	ns
5 kDa vs 25 kDa	0.01461	*
5 kDa vs 40 kDa	0.00057	***
10 kDa vs 25 kDa	0.00329	**
10 kDa vs 40 kDa	0.00013	***
25 kDa vs 40 kDa	0.4928	ns
**0.05**		
5 kDa vs 10 kDa	0.00027	***
5 kDa vs 25 kDa	<0.0001	*****
5 kDa vs 40 kDa	<0.0001	*****
10 kDa vs 25 kDa	<0.0001	*****
10 kDa vs 40 kDa	<0.0001	*****
25 kDa vs 40 kDa	0.52612	ns
**0.1**		
5 kDa vs 10 kDa	<0.0001	*****
5 kDa vs 25 kDa	<0.0001	*****
5 kDa vs 40 kDa	<0.0001	*****
10 kDa vs 25 kDa	<0.0001	*****
10 kDa vs 40 kDa	<0.0001	*****
25 kDa vs 40 kDa	0.69225	ns

Supporting Table S1: Comprehensive p values for Catalase like activity of the different Dex-CeNP formulations.

Tukey's multiple comparisons test		
**0.01**	**P Value**	**Summary**
5 kDa vs 10 kDa	<0.0001	*****
5 kDa vs 25 kDa	<0.0001	*****
5 kDa vs 40 kDa	<0.0001	*****
10 kDa vs 25 kDa	0.00389	**
10 kDa vs 40 kDa	0.00027	***
25 kDa vs 40 kDa	0.64858	ns
**0.05**		
5 kDa vs 10 kDa	<0.0001	*****
5 kDa vs 25 kDa	<0.0001	*****
5 kDa vs 40 kDa	<0.0001	*****
10 kDa vs 25 kDa	0.03786	*
10 kDa vs 40 kDa	<0.0001	*****
25 kDa vs 40 kDa	0.00003	****
**0.1**		
5 kDa vs 10 kDa	<0.0001	*****
5 kDa vs 25 kDa	<0.0001	*****
5 kDa vs 40 kDa	<0.0001	*****
10 kDa vs 25 kDa	0.09515	ns
10 kDa vs 40 kDa	0.00008	****
25 kDa vs 40 kDa	0.01972	*

Supporting Table S2: Comprehensive p values for SOD mimetic like activity of the different Dex-CeNP formulations.

Tukey's multiple comparisons test		
**5 kDa**	**P Value**	**Summary**
Ctrl vs. 0.1	0.9588	ns
Ctrl vs. 0.5	>0.9999	ns
Ctrl vs. 1.0	0.9167	ns
0.1 vs. 0.5	0.9671	ns
0.1 vs. 1.0	0.9989	ns
0.5 vs. 1.0	0.9294	ns
**10 kDa**		

Ctrl vs. 0.1	>0.9999	ns
Ctrl vs. 0.5	>0.9999	ns
Ctrl vs. 1.0	>0.9999	ns
0.1 vs. 0.5	>0.9999	ns
0.1 vs. 1.0	>0.9999	ns
0.5 vs. 1.0	>0.9999	ns
**25 kDa**		
Ctrl vs. 0.1	0.9991	ns
Ctrl vs. 0.5	>0.9999	ns
Ctrl vs. 1.0	>0.9999	ns
0.1 vs. 0.5	0.9991	ns
0.1 vs. 1.0	0.9991	ns
0.5 vs. 1.0	>0.9999	ns
**40 kDa**		
Ctrl vs. 0.1	>0.9999	ns
Ctrl vs. 0.5	>0.9999	ns
Ctrl vs. 1.0	>0.9999	ns
0.1 vs. 0.5	>0.9999	ns
0.1 vs. 1.0	>0.9999	ns
0.5 vs. 1.0	>0.9999	ns

Supporting Table S3: Comprehensive p values on the viability of macrophages (RAW 264.7) for Dex-CeNP formulations.

**Tukey's multiple comparisons test**		
**5 kDa**	**P Value**	**Summary**
Ctrl vs. 0.1	0.9999	ns
Ctrl vs. 0.5	0.9993	ns
Ctrl vs. 1.0	0.9886	ns
0.1 vs. 0.5	>0.9999	ns
0.1 vs. 1.0	0.9942	ns
0.5 vs. 1.0	0.9973	ns
**10 kDa**		
Ctrl vs. 0.1	>0.9999	ns
Ctrl vs. 0.5	>0.9999	ns
Ctrl vs. 1.0	>0.9999	ns
0.1 vs. 0.5	>0.9999	ns

0.1 vs. 1.0	>0.9999	ns
0.5 vs. 1.0	>0.9999	ns
**25 kDa**		
Ctrl vs. 0.1	>0.9999	ns
Ctrl vs. 0.5	>0.9999	ns
Ctrl vs. 1.0	>0.9999	ns
0.1 vs. 0.5	>0.9999	ns
0.1 vs. 1.0	>0.9999	ns
0.5 vs. 1.0	>0.9999	ns
**40 kDa**		
Ctrl vs. 0.1	0.8795	ns
Ctrl vs. 0.5	0.1174	ns
Ctrl vs. 1.0	0.2819	ns
0.1 vs. 0.5	0.4115	ns
0.1 vs. 1.0	0.7037	ns
0.5 vs. 1.0	0.9617	ns

Supporting Table S4: Comprehensive p values on the viability of colon epithelial cells (C2BBe1) for Dex-CeNP formulations.

**Tukey's multiple comparisons test**		
**5 kDa**	**P Value**	**Summary**
Ctrl vs. 0	<0.0001	****
Ctrl vs. 0.1	>0.9999	ns
Ctrl vs. 0.5	0.1941	ns
Ctrl vs. 1.0	>0.9999	ns
0 vs. 0.1	<0.0001	****
0 vs. 0.5	<0.0001	****
0 vs. 1.0	<0.0001	****
0.1 vs. 0.5	0.2438	ns
0.1 vs. 1.0	>0.9999	ns
0.5 vs. 1.0	0.1941	ns
**10 kDa**		
Ctrl vs. 0	<0.0001	****
Ctrl vs. 0.1	<0.0001	****
Ctrl vs. 0.5	0.0009	***
Ctrl vs. 1.0	0.0034	**

0 vs. 0.1	0.1856	ns
0 vs. 0.5	0.0011	**
0 vs. 1.0	0.0003	***
0.1 vs. 0.5	0.2790	ns
0.1 vs. 1.0	0.1177	ns
0.5 vs. 1.0	0.9905	ns
**10 kDa**		
Ctrl vs. 0	<0.0001	****
Ctrl vs. 0.1	0.7122	ns
Ctrl vs. 0.5	0.9686	ns
Ctrl vs. 1.0	0.7169	ns
0 vs. 0.1	<0.0001	****
0 vs. 0.5	<0.0001	****
0 vs. 1.0	<0.0001	****
0.1 vs. 0.5	0.9686	ns
0.1 vs. 1.0	>0.9999	ns
0.5 vs. 1.0	0.9700	ns
**40 kDa**		
Ctrl vs. 0	<0.0001	****
Ctrl vs. 0.1	<0.0001	****
Ctrl vs. 0.5	0.0007	***
Ctrl vs. 1.0	0.0012	**
0 vs. 0.1	0.0185	*
0 vs. 0.5	0.0014	**
0 vs. 1.0	0.0008	***
0.1 vs. 0.5	0.8910	ns
0.1 vs. 1.0	0.8008	ns
0.5 vs. 1.0	0.9996	ns
**Comparison per formulation**		
**Control**	**P Value**	**Summary**
5 kDa vs 10 kDa	>0.9999	ns
5 kDa vs 25 kDa	>0.9999	ns
5 kDa vs 40 kDa	>0.9999	ns
10 kDa vs 25 kDa	>0.9999	ns
10 kDa vs 40 kDa	>0.9999	ns
25 kDa vs 40 kDa	>0.9999	ns
**0**		

5 kDa vs 10 kDa	>0.9999	ns
5 kDa vs 25 kDa	>0.9999	ns
5 kDa vs 40 kDa	>0.9999	ns
10 kDa vs 25 kDa	>0.9999	ns
10 kDa vs 40 kDa	>0.9999	ns
25 kDa vs 40 kDa	>0.9999	ns
**0.01**		
5 kDa vs 10 kDa	<0.0001	****
5 kDa vs 25 kDa	0.9997	ns
5 kDa vs 40 kDa	0.0009	***
10 kDa vs 25 kDa	0.0013	**
10 kDa vs 40 kDa	>0.9999	ns
25 kDa vs 40 kDa	0.0245	*
**0.05**		
5 kDa vs 10 kDa	0.9998	ns
5 kDa vs 25 kDa	0.8303	ns
5 kDa vs 40 kDa	0.9846	ns
10 kDa vs 25 kDa	0.0623	ns
10 kDa vs 40 kDa	>0.9999	ns
25 kDa vs 40 kDa	0.0502	ns
**0.1**		
5 kDa vs 10 kDa	0.0403	*
5 kDa vs 25 kDa	0.9989	ns
5 kDa vs 40 kDa	0.0160	*
10 kDa vs 25 kDa	0.5126	ns
10 kDa vs 40 kDa	>0.9999	ns
25 kDa vs 40 kDa	0.2992	ns

Supporting Table S5: Comprehensive p values for *In vitro* assessment of anti-inflammatory activity by MTS.

**Tukey's multiple comparisons test**		
**5 kDa**	**P Value**	**Summary**
0 vs. 0.1	0.4289	ns
0 vs. 0.5	<0.0001	****
0 vs. 1	<0.0001	****

0.1 vs. 0.5	0.0137	*
0.1 vs. 1	<0.0001	****
0.5 vs. 1	0.3132	ns
**10 kDa**		
0 vs. 0.1	0.5882	ns
0 vs. 0.5	0.0002	***
0 vs. 1	0.0003	***
0.1 vs. 0.5	0.0310	*
0.1 vs. 1	0.0362	*
0.5 vs. 1	>0.9999	ns
**10 kDa**		
0 vs. 0.1	0.0872	ns
0 vs. 0.5	0.0836	ns
0 vs. 1	0.0005	***
0.1 vs. 0.5	>0.9999	ns
0.1 vs. 1	0.3578	ns
0.5 vs. 1	0.3667	ns
**40 kDa**		
0 vs. 0.1	0.1538	ns
0 vs. 0.5	0.0314	*
0 vs. 1	0.0016	**
0.1 vs. 0.5	0.9258	ns
0.1 vs. 1	0.4165	ns
0.5 vs. 1	0.7871	ns
**Comparison per formulation**		
**0**	**P Value**	**Summary**
5 kDa vs 10 kDa	>0.9999	ns
5 kDa vs 25 kDa	>0.9999	ns
5 kDa vs 40 kDa	>0.9999	ns
10 kDa vs 25 kDa	>0.9999	ns
10 kDa vs 40 kDa	>0.9999	ns
25 kDa vs 40 kDa	>0.9999	ns
**0.01**		
5 kDa vs 10 kDa	>0.9999	ns
5 kDa vs 25 kDa	>0.9999	ns
5 kDa vs 40 kDa	>0.9999	ns

10 kDa vs 25 kDa	0.9996	ns
10 kDa vs 40 kDa	>0.9999	ns
25 kDa vs 40 kDa	>0.9999	ns
**0.05**		
5 kDa vs 10 kDa	>0.9999	ns
5 kDa vs 25 kDa	0.5628	ns
5 kDa vs 40 kDa	0.7962	ns
10 kDa vs 25 kDa	0.5274	ns
10 kDa vs 40 kDa	0.2738	ns
25 kDa vs 40 kDa	>0.9999	ns
**0.1**		
5 kDa vs 10 kDa	0.5901	ns
5 kDa vs 25 kDa	0.4923	ns
5 kDa vs 40 kDa	0.2846	ns
10 kDa vs 25 kDa	>0.9999	ns
10 kDa vs 40 kDa	>0.9999	ns
25 kDa vs 40 kDa	>0.9999	ns

Supporting Table S6: Comprehensive p values for *In vitro* assessment of anti-inflammatory activity by Elisa.

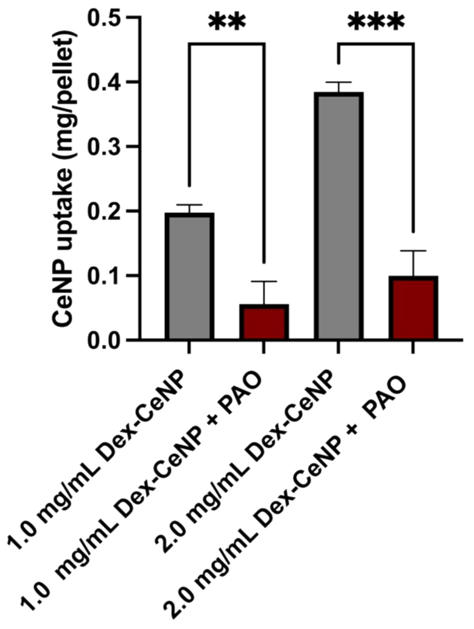

Figure S3: Macrophage cellular uptake. Phenylarsine oxide, an inhibitor of clathrin mediated endocytosis, reduces Dex-CeNP uptake in macrophages.

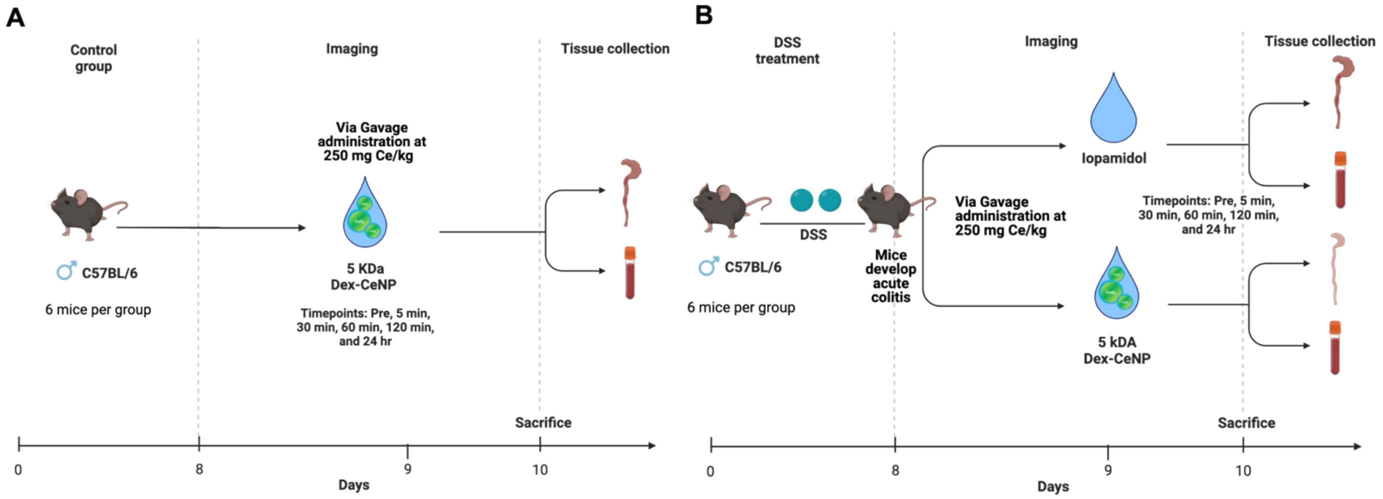

Figure S4: Schematic overview of experimental procedures to evaluate contrast agent performance. (**A**) Control group, (**B**) Colitis group.

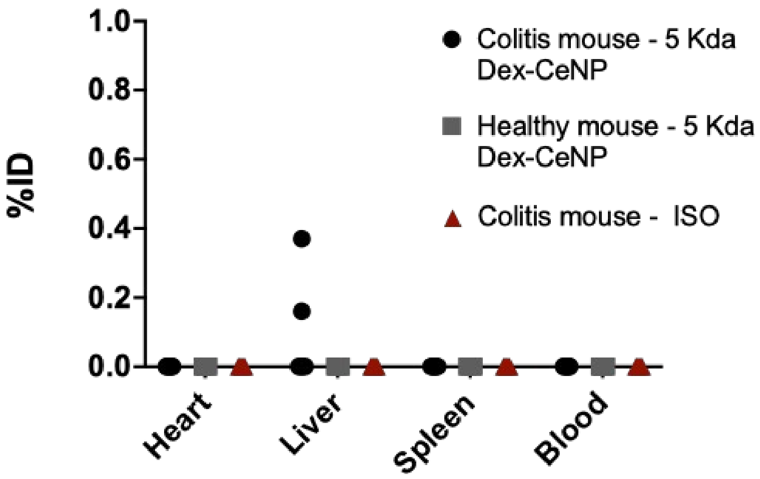

Figure S5: Biodistribution of Dex-CeNP in healthy and DSS-colitis mice at 24 hours post administration in the heart, liver, spleen and blood.

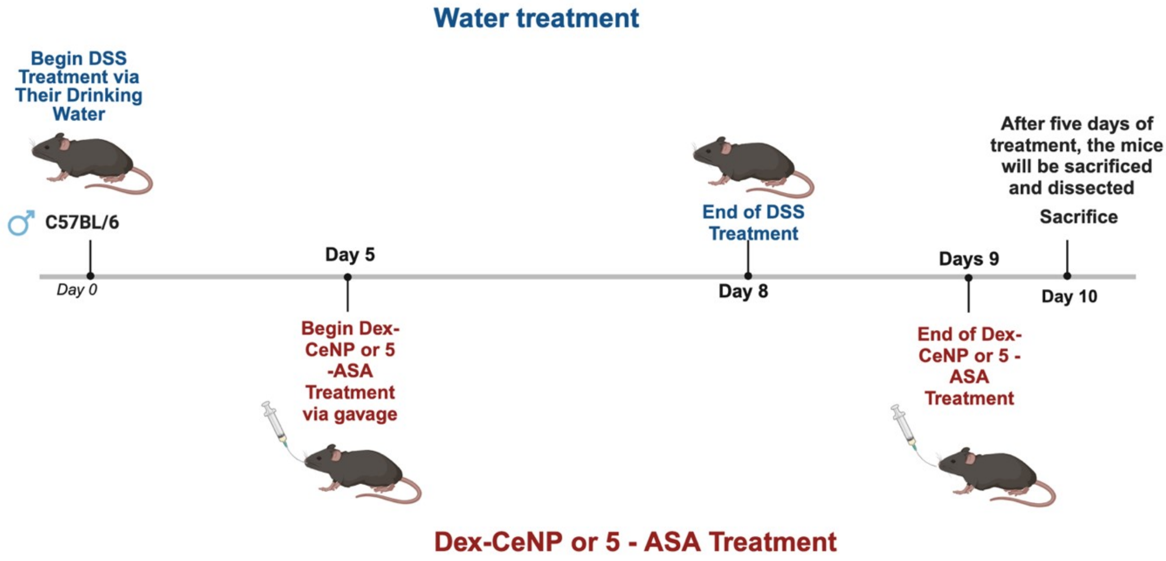

Figure S6: Schematic overview of experimental procedures to evaluate therapeutic performance.

**Tukey's multiple comparisons test**		
**Comparison**	**P Value**	**Summary**
Water vs Dex-CeNP	.115	ns
Water vs 5 - ASA	.823	ns
5 kDa Dex-CeNP vs 5 - ASA	.194	ns

Supporting Table S7: Comprehensive p-values for differences in colon length between groups.

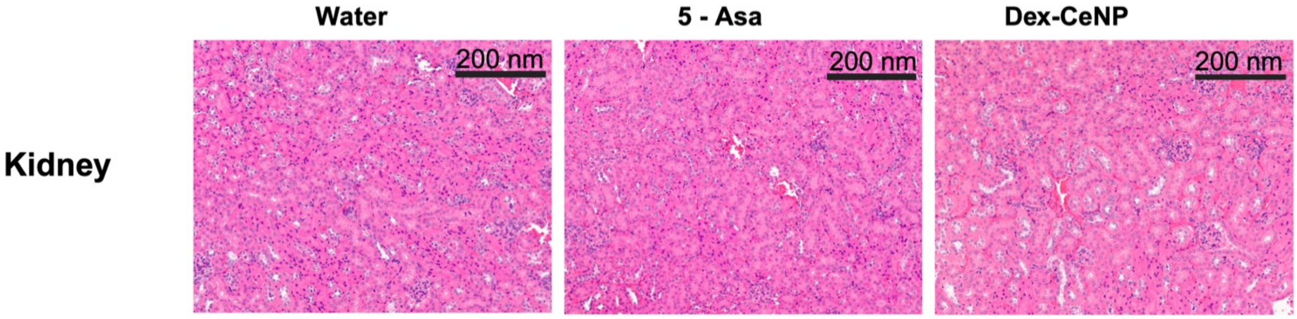

Figure S7: Micrographs of H&E-stained of kidney.

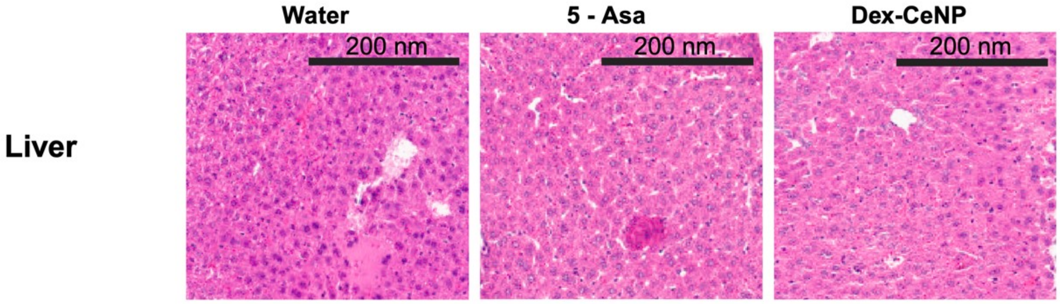

Figure S8: Micrographs of H&E-stained of liver.

**DSS/Mucosal/Crypt Loss:**	Normal mucosa	0
	Shortening of basal one-third of crypts +/- slight inflammation and edema in lamina propria.	1
	Loss of basal two-thirds of crypts +/- moderate inflammation in lamina propria.	2
	Loss of all epithelium +/- severe inflammation in lamina propria +/- submucosa inflammation but with surface epithelium still remaining.	3

	Loss of all epithelium, including surface epithelium +/- severe inflammation in the lamina propria and submucosa +/- muscularis. An exudate containing cell debris, inflammatory cells, fibrin and mucus covers the damaged mucosa.	4
**Crypt Inflammation:**	Normal	0
	1-2 inflammatory cells	1
	Cryptitis	2
	Crypt abscess/dirty necrosis	3
**Lamina Propria Mononuclear Cells:**	Normal	0
	Slight increase	1
	Moderate increase	2
	Marked increase	3
**Neutrophils:**	Normal	0
	Slight increase	1
	Moderate increase	2
	Marked increase	3
**Epithelial hyperplasia:**	Normal	0
	Mild	1
	Moderate increase	2
	Discrete nest of regenerated crypts delineated from adjacent mucosa with no obvious disruption of overlying mucosal surface	3
**Edema/fibrosis:**	None	0
	Mild/focal/single layer of colon	1
	Moderate/multifocal/multiple layers	2
	Severe/widespread/transmural	3
**Maximum Total:**		19

Supporting Table S8: Histopathological Inflammation Scoring System for Disease Severity Assessment.
